# Lactate-to-albumin ratio improves prediction of perioperative mortality in lung transplant patients

**DOI:** 10.3389/fmed.2026.1837904

**Published:** 2026-08-18

**Authors:** Yu Yi, Ge Luo, Ying Wang, Xinhong Ran, Xinchen Tao, Qiuping Wang, Qi Gao, Yuanyuan Yao, Min Yan

**Affiliations:** 1 Department of Anesthesiology, The Second Affiliated Hospital, Zhejiang University School of Medicine, Hangzhou, China; 2 Department of Anesthesiology, Suzhou Xiangcheng People’s Hospital, Suzhou, China

**Keywords:** lactate-to-albumin ratio, lung transplantation, mortality, nomogram, prediction model

## Abstract

**Backgrounds:**

Perioperative mortality remains elevated among lung transplant recipients. The lactate-to-albumin ratio (LAR) is a prognostic indicator, but its role in predicting mortality in lung transplant patients remains unclear. The study aims to assess the incremental prognostic value of LAR and develop a more accurate prediction model for perioperative mortality in lung transplant patients.

**Methods:**

This study conducted a single-center, retrospective analysis involving 379 lung transplant recipients. A base model was developed to predict 30-day postoperative mortality and a modified model was then constructed by incorporating LAR. Model performance was evaluated using the area under the curve (AUC), Brier score, and calibration curves. DeLong and likelihood ratio tests compared models, while continuous Net Reclassification Improvement (NRI) and Integrated Discrimination Improvement (IDI) quantified accuracy improvements.

**Results:**

Of 379 patients, 51 (13.46%) died within 30 days post-transplant. LAR was found to be an independent predictor of perioperative mortality in lung transplant patients. Other predictors in the modified model included blood transfusion, cold ischemia time, and fluid balance. The inclusion of LAR significantly improved the modified model’s discriminatory ability (AUC: 0.807 vs. 0.764; *p* = 0.024) and calibration (Brier score: 0.0927 vs. 0.1025). The likelihood ratio test confirmed a significantly improved model fit (*p* < 0.0001). The continuous NRI and IDI were 0.643 and 0.090, respectively (*p* < 0.001). A nomogram and an interactive web-based tool were developed to facilitate individualized risk assessment.

**Conclusion:**

The modified model incorporating LAR provides a more accurate and comprehensive assessment of perioperative mortality risk.

## Introduction

Globally, approximately 4,000 lung transplantations are performed annually, offering significant improvements in survival duration and quality of life for individuals with end-stage lung disease (1). Notwithstanding advancements in allocation systems, surgical techniques and perioperative management, lung transplantation (LTx) recipients continue to experience high mortality risk (1). Developing a robust predictive model for postoperative mortality is crucial to identifying high-risk patients, optimizing clinical decision-making, and improving post-transplant outcomes through targeted interventions and personalized care strategies.

Several predictive tools, including the preoperative Risk Stratification Score (RSS), Prognostic Nutritional Index (PNI), and the MALT score, have been proposed to identify patients at elevated risk of perioperative mortality (2–4). Although these tools incorporate multiple clinical and laboratory parameters, their clinical utility remains constrained due to limited predictive performance. Current predictive models for mortality in LTx predominantly focus on postoperative parameters, such as ICU stay duration, the occurrence of 72-h primary graft dysfunction grade 3 (PGD3), and postoperative extracorporeal membrane oxygenation (ECMO) duration (5). These limitations often hinder timely clinical interventions, highlighting the need for predictive tools that integrate early perioperative variables.

Lactate, a byproduct of anaerobic metabolism, reflects tissue hypoperfusion and cellular hypoxia and has been associated with organ failure and increased mortality in critically ill patients (6, 7). Serum albumin, an established marker of nutritional status, functions as a negative acute-phase reactant during inflammation and is correlated with the severity of inflammatory responses, disease prognosis, and mortality (8–10). The lactate-to-albumin ratio (LAR), which reflects tissue perfusion, inflammatory response, immune function, and nutritional status, can improve the accuracy of models for predicting mortality risk. Previous studies have established a strong association between LAR and mortality in patients with severe sepsis, myocardial infarction, and respiratory failure, highlighting its potential as an early prognostic marker (9, 11, 12).

Despite these findings, the predictive value between LAR and perioperative mortality following lung transplantation has not been investigated. This study seeks to evaluate the incremental prognostic value of LAR and develop a more accurate prediction model for perioperative mortality in lung transplant patients.

## Methods

### Setting and study population

Clinical data were collected from all patients who underwent lung transplantation at a tertiary hospital between January 2021 and December 2023. This study was conducted in accordance with the principles of the Declaration of Helsinki and received approval from the Ethics Committee of the Second Affiliated Hospital, Zhejiang University School of Medicine (Reference number: 2022 0352). All personal information in the collected data was de-identified, and the requirement for informed consent was waived by the Ethics Committee.

### Patient eligibility

The study population included patients who underwent lung transplantation. Exclusion criteria were as follows: (1) patients who underwent multi-organ transplantation or re-transplantation; (2) patients under 18 years of age; and (3) patients with missing essential data, specifically 30-day mortality or LAR.

### Endpoint

The primary endpoint of the study was 30-day mortality, defined as all-cause death within 30 days after lung transplantation.

### Data extraction

The variables in this study were categorized as (i) demographic characteristics; (ii) preoperative characteristics; (iii) intraoperative characteristics; (iv) postoperative characteristics; and (v) donor characteristics. Demographic characteristics included patient sex, age, and body mass index (BMI). Preoperative characteristics encompassed primary diagnosis, comorbidities (cardiac dysfunction, pulmonary hypertension, diabetes), ASA classification, prior surgeries, preoperative tracheal intubation, ECMO use, and laboratory results. Intraoperative characteristics included surgical duration, transplantation type, fluid balance (calculated as intake minus output) (29), blood transfusions, blood loss, albumin infusion, norepinephrine dose, intraoperative hemodialysis, and ECMO type. Postoperative characteristics included ECMO use, LAR on the first ICU day, and CRP levels. Donor characteristics included donor sex, age, ventilation duration, PaO_2_/FiO_2_, cold ischemia time, and donor type.

### Statistical analysis

Demographic, preoperative, intraoperative, postoperative, and donor characteristics were analyzed using appropriate statistical methods. Missing data were addressed with multiple imputation by chained equations. Univariate logistic regression identified potential predictors of perioperative mortality, and significant variables (*p* < 0.05) were included in a multivariate logistic regression model. Two models—a base model (excluding LAR) and a modified model (including LAR)—were compared using AUC, the DeLong test, calibration curves, and the Hosmer–Lemeshow test. Internal validation was conducted via bootstrap resampling, and decision curve analysis assessed clinical utility. All analyses were performed in RStudio (version 4.3.3), with *p* < 0.05 considered significant.

Details on imputation, spline analysis, multicollinearity, discrimination improvement, bootstrap resampling, calibration, and nomogram development are provided in the Supplementary material.

## Results

Data from 398 adult patients who underwent lung transplantation were analyzed. Eleven participants were excluded due to multi-organ transplantation or re-transplantation (Supplementary Figure S1). The distribution of missing data is illustrated in Supplementary Figure S2, accounting for only 5.77%. After excluding patients with missing LAR data or 30-day mortality information, 379 patients were included in the final analysis. A restricted cubic spline function was employed to examine nonlinear relationships among variables and to determine whether continuous variables required categorization. Surgery duration was the only variable exhibiting a significant nonlinear relationship with perioperative mortality, as shown in Supplementary Figure S3.

### Patient characteristics and predictors of perioperative mortality

Among the 379 eligible patients, 51 (13.46%) died within 30 days following lung transplantation, while 328 survived. Table 1 provides a detailed summary of recipient preoperative, intraoperative, and postoperative characteristics, donor characteristics, and 30-day postoperative survival outcomes. Patients who died within 30 days were more likely to have undergone surgeries exceeding 321 min (*p* < 0.05). Increased norepinephrine doses and positive fluid balance were also significantly associated with higher 30-day mortality risk (*p* < 0.05). Additionally, patients requiring greater blood and albumin transfusions exhibited a higher likelihood of postoperative mortality (*p* < 0.05). Elevated LAR and prolonged cold ischemia time further correlated with an increased risk of 30-day mortality (p < 0.05).

**Table 1:** Study group characteristics and univariable analysis results for perioperative mortality after lung transplantation.

Characteristics	Survivors	Non-survivors	Univariate analysis *p* value
	(*N* = 328)	(*N* = 51)	
Preoperative characteristics			
Demographic information			
Sex, *n* (%)			0.222
Male	277 (84.5%)	39 (76.5%)	
Female	51 (15.5%)	12 (23.5%)	
Age (year)	59.00 (52.00, 66.00)	59.00 (52.50, 66.00)	0.897
BMI, kg/m^2^	20.90 (17.70, 23.80)	21.10 (17.40, 23.80)	0.841
Diagnosis, *n* (%)			0.933
Chronic obstructive pulmonary diseases	69 (21.0%)	13 (25.5%)	
Interstitial pulmonary fibrosis	166 (50.6%)	25 (49.0%)	
Pneumoconiosis	46 (14.0%)	6 (11.8%)	
Cystic fibrosis	18 (5.5%)	2 (3.9%)	
Others	29 (8.8%)	5 (9.8%)	
Comorbidities			
Cardiac dysfunction, *n* (%)			0.919
No	284 (86.6%)	45 (88.2%)	
Yes	44 (13.4%)	6 (11.8%)	
Respiratory failure, *n* (%)			0.485
No	84 (25.6%)	16 (31.4%)	
Yes	244 (74.4%)	35 (68.6%)	
Pulmonary hypertension, *n* (%)			0.097
No	142 (43.3%)	29 (56.9%)	
Yes	186 (56.7%)	22 (43.1%)	
Hypertension, *n* (%)			0.595
No	283 (86.3%)	42 (82.4%)	
Yes	45 (13.7%)	9 (17.6%)	
Autoimmune comorbidity, *n* (%)			0.591
No	289 (88.1%)	43 (84.3%)	
Yes	39 (11.9%)	8 (15.7%)	
Diabetes mellitus, *n* (%)			0.198
No	276 (84.1%)	47 (92.2%)	
Yes	52 (15.9%)	4 (7.8%)	
Preoperative conditions			
ASA classification, *n* (%)			0.504
III	40 (12.2%)	4 (7.8%)	
≥IV	288 (87.8%)	47 (92.2%)	
Medical condition, *n* (%)			0.828
Ward	221 (67.4%)	33 (64.7%)	
ICU	107 (32.6%)	18 (35.3%)	
Previous surgeries, *n* (%)			>0.99
No	229 (69.8%)	36 (70.6%)	
Yes	99 (30.2%)	15 (29.4%)	
Preoperative tracheal intubation, *n* (%)			0.519
No	249 (75.9%)	36 (70.6%)	
Yes	79 (24.1%)	15 (29.4%)	
Preoperative ECMO, *n* (%)			0.192
No	256 (78.0%)	35 (68.6%)	
Yes	72 (22.0%)	16 (31.4%)	
Preoperative laboratory results			
Haemoglobin (g/dl)	129 (112, 141)	128 (108, 139)	0.488
Lactate (mmol/L)	1.60 (1.20, 2.20)	1.80 (1.35, 2.40)	0.103
Albumin (g/L)	35.80 (32.80, 38.90)	35.40 (32.30, 37.70)	0.259
CRP (mg/L)	9.20 (3.55, 26.90)	8.65 (4.45, 30.80)	0.476
Serum creatinine (umol/L)	62.10 (50.00, 73.00)	61.00 (49.50, 76.10)	0.893
Intraoperative characteristics			
Duration of surgery, *n* (%)			<0.001*
<321 min	179 (54.6%)	14 (27.5%)	
>321 min	149 (45.4%)	37 (72.5%)	
Type of lung transplantation, *n* (%)			0.203
Single lung transplantation	124 (37.8%)	14 (27.5%)	
Double lung transplantation	204 (62.2%)	37 (72.5%)	
Norepinephrine dose (mg)	4.00 (1.20, 8.19)	6.00 (2.00, 18.00)	0.008*
Fluid balance (L)	0.240 (−0.35, 1.21)	1.58 (0.28, 2.78)	<0.001*
Blood transfusion (units)	0.35 (0, 4.00)	4.00 (0, 11.30)	<0.001*
Blood loss (L)	0.75 (0.50, 1.20)	1.30 (0.70, 3.25)	<0.001*
Total albumin infusion (g)	110 (80.00, 150)	129 (101, 154)	0.026*
Intraoperative hemodialysis, *n* (%)			>0.99
No	310 (94.5%)	48 (94.1%)	
Yes	18 (5.5%)	3 (5.9%)	
Intraoperative ECMO type, *n* (%)			0.556
No	27 (8.2%)	2 (3.9%)	
Venovenous	250 (76.2%)	41 (80.4%)	
Venoarterial	51 (15.5%)	8 (15.7%)	
Postoperative characteristics			
Postoperative ECMO, *n* (%)			0.251
No	33 (10.1%)	2 (3.9%)	
Yes	295 (89.9%)	49 (96.1%)	
Postoperative ECMO type, *n* (%)			0.275
No	33 (10.1%)	2 (3.9%)	
Venovenous	274 (83.5%)	44 (86.3%)	
Venoarterial	21 (6.4%)	5 (9.8%)	
Lactate-to-albumin ratio	0.49 (0.32, 0.87)	1.33 (0.53, 2.66)	<0.001*
Postoperative CRP (mg/L)	11.30 (5.60, 27.70)	9.30 (5.70, 21.80)	0.365
Donor characteristics			
Sex, *n* (%)			0.561
Female	55 (16.8%)	6 (11.8%)	
Male	268 (81.7%)	42 (82.4%)	
Age (y)	60.00 (52.00, 66.00)	58.00 (51.00, 66.00)	0.567
Mechanical ventilation time (h)	7.00 (4.00, 10.00)	8.00 (5.00, 11.00)	0.080
PaO_2_/FiO_2_ (mmHg)	403 (342, 457)	409 (355, 486)	0.493
Cold ischemia time (h)	7.50 (6.20, 8.00)	7.90 (6.80, 9.00)	0.017*
Type of donor, *n* (%)			0.751
DBD	312 (95.1%)	50 (98.0%)	
DCD	13 (4.0%)	1 (2.0%)	

Data are presented as Median (IQR) or *n* (%).

BMI, Body mass index; ICU, Intensive care unit; ECMO, extracorporeal membrane oxygenation; CRP, C-reactive protein; PaO /FiO_2_, arterial oxygen tension/inspired oxygen fraction; DCD: Donation after Circulatory Death; DBD: Donation after Brain Death; IQR, Interquartile range.

*Significant values.

### Development of perioperative mortality prediction models

Univariate analysis identified surgery duration, total albumin infusion, fluid balance, blood transfusion, blood loss, norepinephrine, LAR, and cold ischemia time as potential predictors of perioperative mortality. Blood loss was excluded due to a strong correlation with blood transfusion, as indicated in Supplementary Figure S4 (*r* = 0.85, *p* < 0.001). Multivariate logistic regression, guided by AIC-based stepwise selection, was performed to develop the base model, excluding LAR. This analysis identified fluid balance, blood transfusion, and cold ischemia time as predictors. The modified model was constructed by incorporating LAR into the base model, resulting in the final predictive models outlined in Table 2. VIFs for the modified model were lower than 5, indicating minimal multicollinearity among variables (Supplementary Table S1). Supplementary Figure S5 shows no significant departure from linearity for the continuous predictors, supporting the assumptions of the multivariable logistic regression model.

**Table 2:** Multivariable logistic regression models for perioperative mortality after lung transplantation.

Variables	Base model		Modified model		
	OR (95%CI)	*p* value	Unadjusted OR (95%CI)	Adjusted OR (95%CI)	*p* value
Fluid balance (l)	1.48 (1.15, 1.91)	0.002	1.68 (1.38, 2.05)	1.43 (1.08,1.87)	0.009
Cold ischemia time (h)	1.30 (1.05, 1.60)	0.011	1.31 (1.07, 1.6)	1.30 (1.04,1.61)	0.015
Blood transfusion (units)	1.06 (1.00, 1.13)	0.061	1.13 (1.08, 1.19)	0.96 (0.89,1.04)	0.366
Lactate-to-albumin ratio	–	–	2.71 (1.97, 3.72)	2.52 (1.65,3.83)	<0.001

OR, Odds ratio; CI, Confidence interval.

### Model performance evaluation of base and modified models

**Figure 1:**
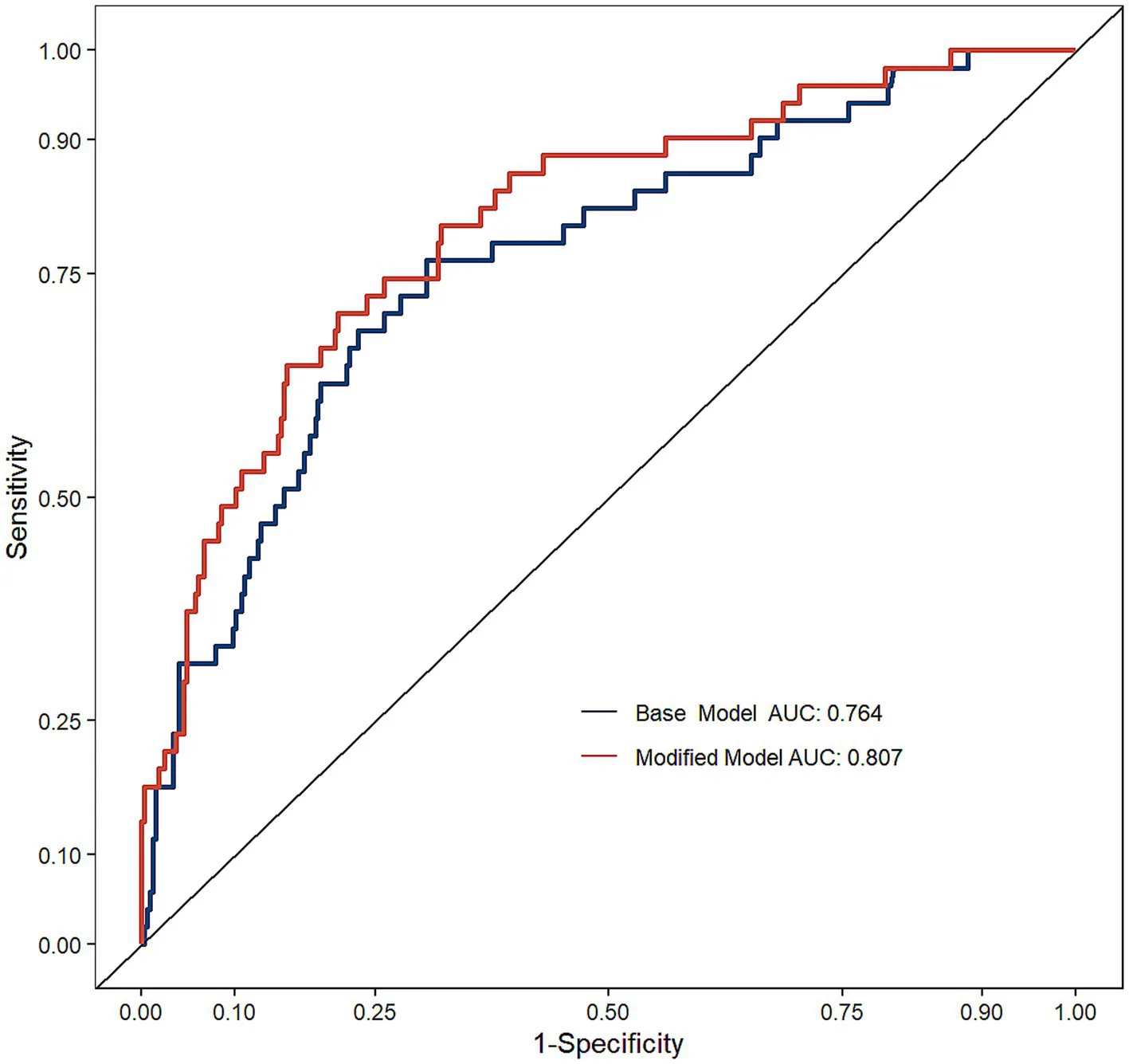
Receiver operating characteristic (ROC) curves for the base model and modified model.

**Figure 2:**
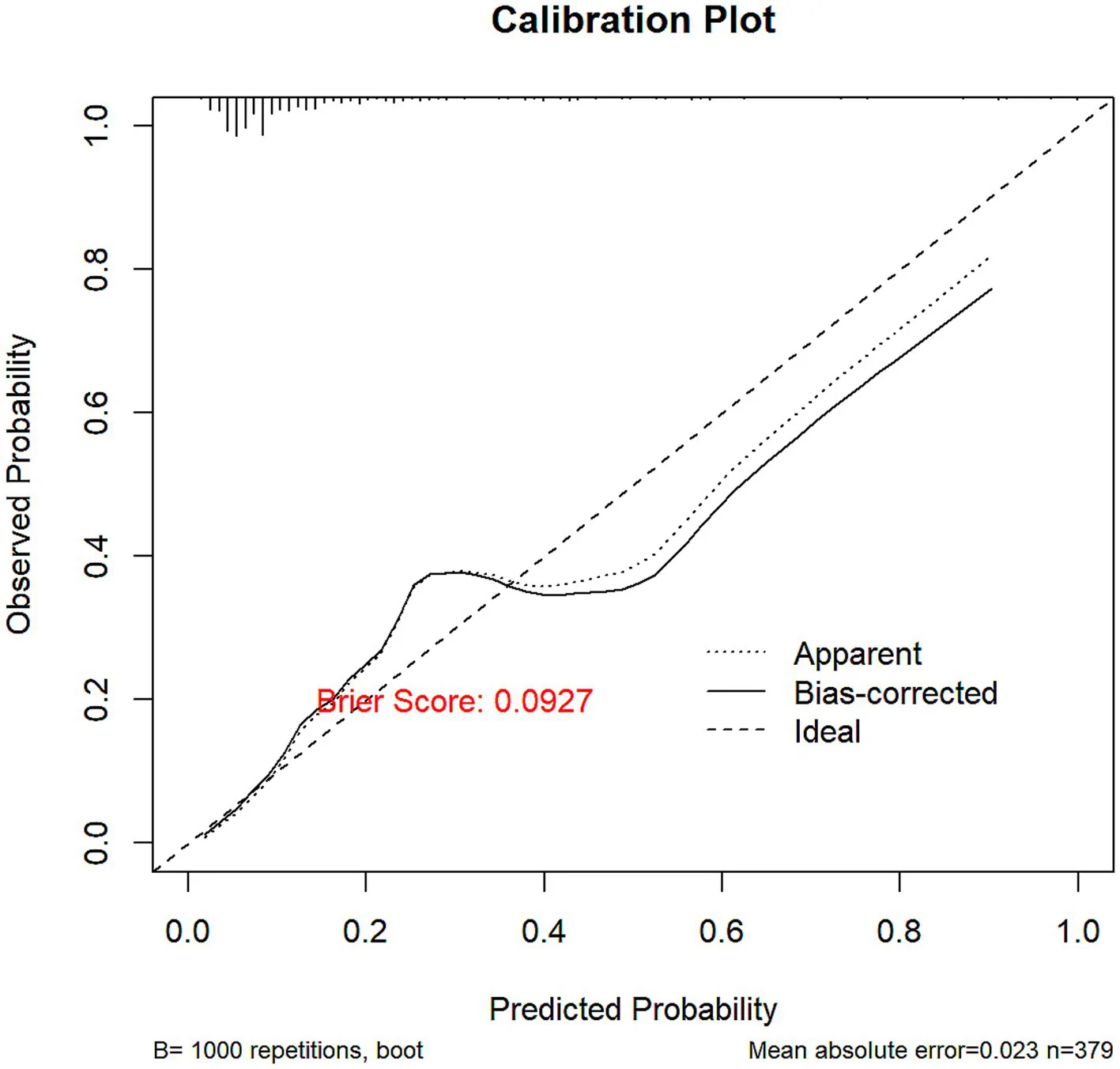
The bootstrap calibration curves for the modified model.

ROC curve analysis demonstrated that the modified model exhibited superior discriminatory ability (Figure 1). The addition of LAR to the base model significantly enhanced predictive performance for perioperative mortality risk in lung transplantation, increasing the AUC from 0.764 (95% CI: 0.6908–0.8363) to 0.807 (95% CI: 0.7413–0.8734; *p* = 0.024, based on the DeLong test). The likelihood ratio test further confirmed a statistically significant improvement in model fit (*p* < 0.0001), indicating that LAR substantially improved the model’s discriminatory capacity and overall predictive performance. Bootstrap validation of the modified model yielded a low optimism value of 0.0136, resulting in a bias-corrected AUC of 0.794, compared to 0.749 for the base model. The Brier score for the modified model also decreased from 0.1025 to 0.0927, reflecting improved prediction accuracy. Calibration curves demonstrated a strong alignment between predicted and observed probabilities, further supporting robust calibration (Figure 2). The Hosmer–Lemeshow test yielded *p*-values above 0.05, supporting the model’s calibration accuracy. Continuous NRI was 0.643 (95% CI: 0.356–0.931), and the IDI was 0.090 (95% CI: 0.036–0.143), both indicating enhanced predictive performance of the modified model (Table 3).

**Table 3:** Comparison of model performance between the base model and the modified model.

Performance metrics	Base model	Modified model
AUC (95% CI)	0.763 (0.691–0.836)	0.807 (0.741–0.873)
Bias-corrected AUC	0.749	0.794
Brier score	0.1025	0.0927
Hosmer–Lemeshow *p*-value	0.5923	0.5442
Continuous NRI (95% CI)	REF	0.643 (0.356–0.931)
IDI (95% CI)	REF	0.0895 (0.036–0.143)

AUC, areas under the receiver operator characteristic curve; IDI, integrated discrimination improvement index; NRI, net reclassification index.

Modified model was base model plus LAR.

### Development and clinical utility of the nomogram

**Figure 3:**
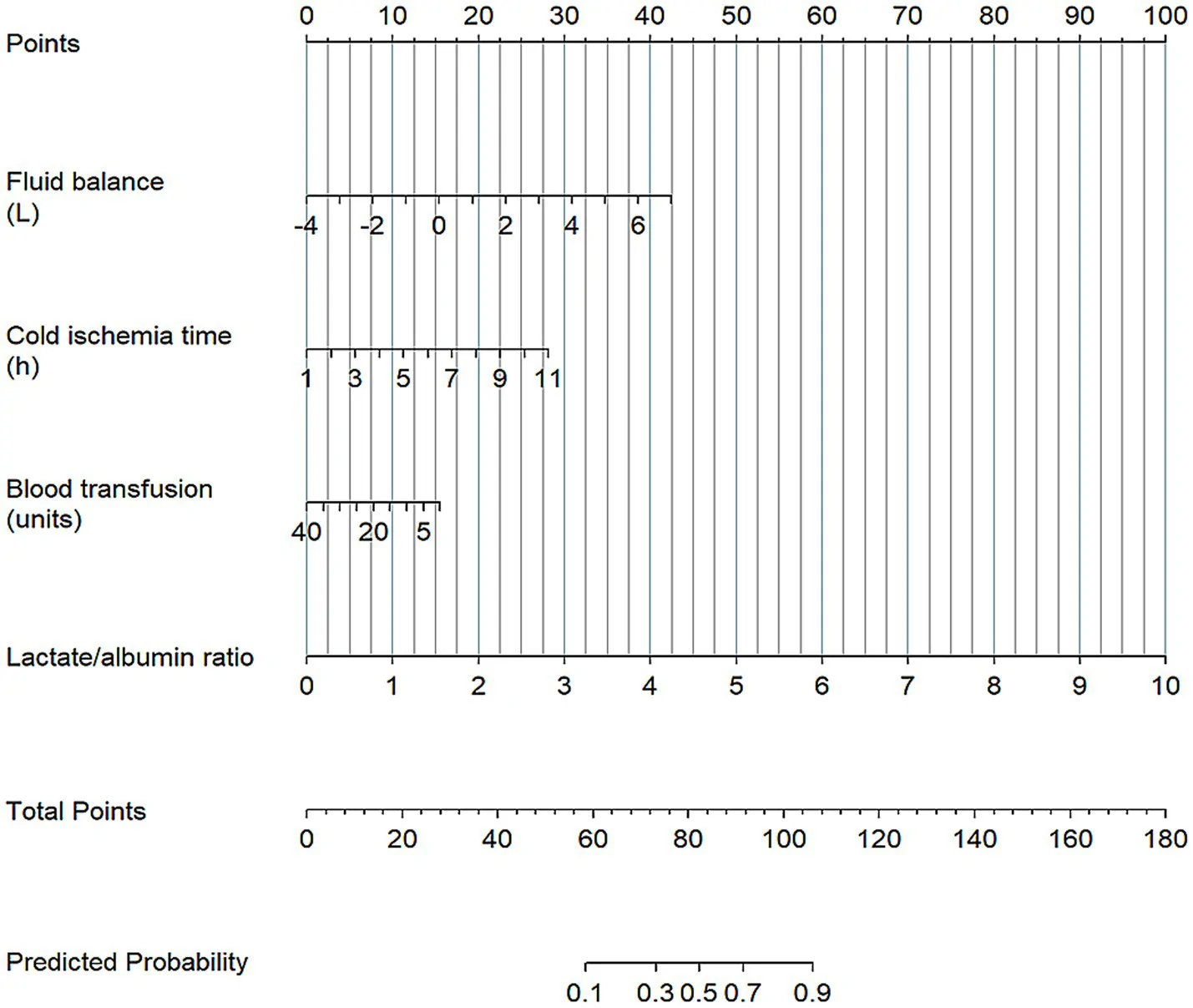
Nomogram predicting perioperative mortality after lung transplantation. For each patient, a vertical line is drawn from the value of each predictor to the “Points” axis to obtain the corresponding score. The scores of all predictors are then summed to generate the “Total Points,” which are projected downward to estimate the predicted probability of 30-day mortality. The nomogram is intended for postoperative risk estimation after completion of intraoperative management. The points assigned to each predictor reflect its contribution within the prediction model and should not be interpreted as the effect of modifying any individual predictor or as guidance for specific clinical management decisions.

**Figure 4:**
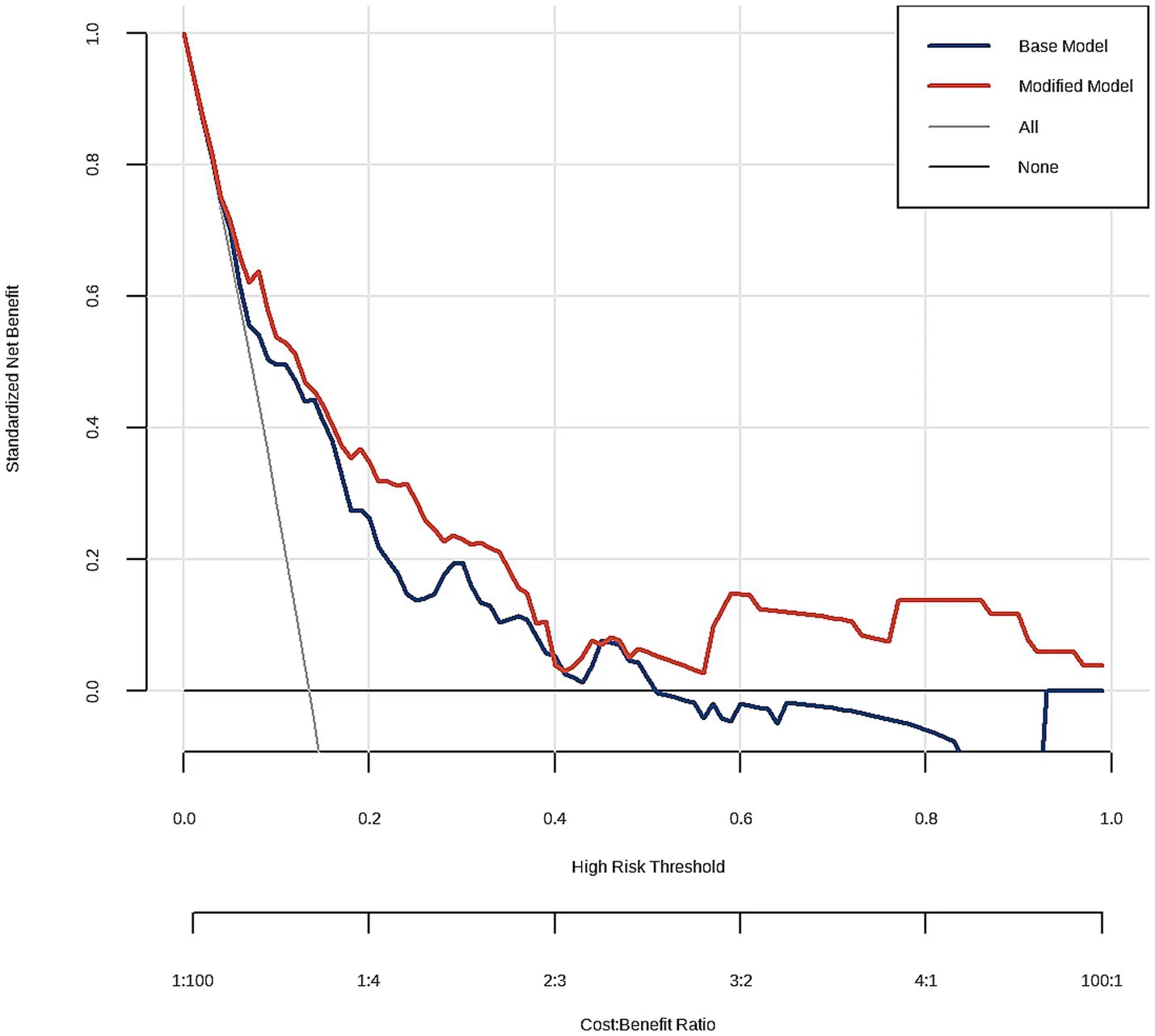
Decision curve analysis for the base model and modified model. The *x*-axis represents the threshold probability at which a clinician would consider a patient to be at high risk and take additional intervention, whereas the *y*-axis represents the standardized net benefit. The gray line represents the strategy of treating all patients as high risk, and the black horizontal line represents the strategy of treating no patients as high risk.

Based on multivariate logistic regression results, a nomogram was developed to visually represent the risk of perioperative mortality in lung transplantation (Figure 3). The nomogram depicted the contribution of each predictor to the overall estimated risk. The points assigned to each predictor reflect its contribution within the prediction model and should not be interpreted as the effect of modifying any individual predictor or as guidance for specific clinical management decisions. A dynamic web-based calculator was also developed to simplify the application of the nomogram and provide a reference range for perioperative mortality probabilities. Further details are available at https://nomogramformortalityafterlungtransplantation.shinyapps.io/dynnomapp/. Decision curve analysis showed that the base model provided clinical benefit within a threshold probability range of 0–0.5, while the modified model demonstrated an extended range of benefit from 0 to 0.99 (Figure 4). Overall, the modified model offered a higher net benefit in predicting perioperative mortality risk in lung transplantation. Additional analyses based on five imputed datasets are presented in Supplementary Table S2.

## Discussion

Two models were developed in this study to predict the 30-day mortality risk following lung transplantation: a base model and a modified model that incorporated the LAR. The findings demonstrated that the LAR-inclusive model considerably outperformed the base model across multiple evaluation metrics. A nomogram based on the modified model was also developed, offering clinicians a practical tool to assess mortality risk, support early postoperative risk stratification, and assist clinical decision-making.

Existing predictive tools for mortality following lung transplantation exhibit limitations that constrain their clinical applicability and accuracy. The RSS uses preoperative recipient variables exclusively to predict mortality. Although it incorporates 11 variables, its AUC is limited to 0.67, reflecting modest discriminatory ability (2). Similarly, the MALT score combines donor and recipient variables but excludes critical perioperative physiological markers, resulting in a constrained AUC of 0.66 (4). The PNI incorporates nutritional indicators but is based on a small sample of 132 patients, which limits its predictive stability and reliability across diverse populations (3). Conversely, several postoperative mortality prediction models rely heavily on postoperative indicators such as ICU stay duration, PGD3, and postoperative ECMO duration (5). While these variables are predictive, they can only be measured after the onset of severe complications, limiting their utility in early intervention. Compared with previously reported models, the modified model offers substantial advantages. The inclusion of LAR significantly improved the model’s predictive accuracy, as evidenced by an AUC increase to 0.807, a reduction in the Brier score, and positive NRI and IDI values. These improvements suggest that the modified model achieved better discrimination, lower prediction error, and improved risk reclassification. Because LAR can be obtained on the first postoperative day, these gains may support early postoperative risk stratification and timely identification of patients at increased risk of mortality. For patients with a high predicted risk, clinicians may consider intensified postoperative surveillance, including closer evaluation of hemodynamic status, tissue perfusion, graft lung function, renal function, and infection-related complications, as well as earlier multidisciplinary assessment when clinically appropriate. Therefore, the modified model may provide supportive information for risk-adapted postoperative management, including adjustment of monitoring intensity, ICU resource allocation, and individualized postoperative care planning.

In recent years, the LAR has gained recognition as a valuable prognostic biomarker in critically ill patients (13–16). Shadvar et al. reported that LAR outperformed traditional indicators in predicting outcomes for patients with septic shock, achieving a significantly higher AUC (13). Similarly, Wang et al. demonstrated the reliability of LAR as a prognostic indicator for infectious diseases, underscoring its broad applicability across diverse patient populations (17). Because lactate and albumin are produced by different organs and regulated through distinct mechanisms, their combination reduces single-factor bias, providing a more comprehensive assessment of metabolic stress and immune response. Elevated lactate levels are indicative of hypoxia and ischemia–reperfusion injury, both of which adversely affect postoperative outcomes (18). For example, patients with impaired cardiac function are more likely to experience elevated lactate levels during surgery, particularly during pulmonary artery clamping and single-lung ventilation, where tissue hypoxia is exacerbated (19). Additionally, lung transplantation triggers a pronounced inflammatory response due to surgical trauma and immune rejection of the transplanted organ. Low albumin levels serve as a marker of heightened inflammatory responses and are strongly associated with poor prognoses (20). By integrating lactate and albumin, LAR offers a holistic reflection of the pathophysiological state in lung transplant patients, enhancing its reliability as a predictor of postoperative mortality risk. This study is the first to incorporate LAR into a perioperative mortality risk prediction model for lung transplantation. Compared with the base model, the inclusion of LAR significantly improved predictive performance, as evidenced by an increase in AUC from 0.763 to 0.807 (DeLong test, *p* = 0.02374). In this study, the likelihood ratio test confirmed that the inclusion of LAR significantly improved model fit (21). Additionally, The Brier score decreased from 0.1025 to 0.0927, while NRI and IDI increased by 0.6434 and 0.0895, highlighting LAR’s contribution to predictive performance. Moreover, LAR is an objective and easily measurable biomarker that effectively identifies high-risk lung transplant patients during the early postoperative period.

In addition to LAR, cold ischemia time (CIT) and perioperative fluid balance were important predictors in the model, each contributing to improved risk assessment of perioperative mortality risk in lung transplant recipients. Postoperative complications, particularly primary graft dysfunction (PGD), represent the primary contributors to early mortality in lung transplant patients (22). A large-scale study demonstrated that each additional liter of intraoperative fluid administration increased the risk of severe PGD by 22% (23). This finding strongly demonstrates the detrimental effects of excessive fluid management on early postoperative outcomes. Consistent with our findings, growing evidence supports the association between prolonged CIT and higher perioperative mortality in lung transplant recipients (24–26). Prolonged CIT has been shown to induce ischemia–reperfusion injury, triggering inflammation and oxidative stress that directly impair graft lung function (27). This injury tends to initiate PGD and further weakens pulmonary immune defenses, increasing susceptibility to infections and exacerbating poor outcomes (28). A large-scale analysis revealed that double-lung recipients with CIT exceeding 6 h exhibited significantly higher 30-day mortality risk (26). These findings, aligned with our study, underscore the critical need to minimize ischemic time as part of strategies to improve survival in lung transplantation.

DCA further highlighted the potential clinical utility of the modified model. Across a relatively broad range of risk thresholds, the modified model generally provided a higher net benefit than the base model, supporting its value in guiding early postoperative risk stratification and perioperative management decisions. However, at higher threshold probabilities, particularly above approximately 0.35, the absolute net benefit of the modified model was relatively limited, suggesting that the incremental clinical benefit within this threshold range may be modest. Therefore, this model should be considered an adjunctive tool to support clinical judgment, particularly for informing postoperative monitoring intensity and supportive care planning, rather than as a substitute for clinical judgment.

### Limitations

Several limitations should be acknowledged. First, the retrospective design of the study introduces selection bias, and the influence of unknown confounding factors cannot be entirely excluded. Additionally pulmonary function test data were unavailable, which may limit the model’s overall performance. Third, the proposed nomogram should be interpreted primarily as a risk stratification tool rather than as a direct guide for personalized therapeutic interventions. Finally, as a single-center study, the broader applicability of the nomogram remains uncertain, despite internal validation through bootstrapping. Therefore, prospective, multicenter studies are warranted in the future to perform external validation and to explore whether this nomogram-guided risk stratification can optimize clinical management and improve outcomes for lung transplant recipients.

## Conclusion

This single-center retrospective study identified the lactate-to-albumin ratio (LAR) as an independent predictor of perioperative mortality in lung transplant patients. The modified model, incorporating LAR, offers a more accurate and comprehensive assessment of perioperative mortality risk.

## Data Availability

The raw data supporting the conclusions of this article will be made available by the authors, without undue reservation.

## Glossary

AUC: areas under the receiver operator characteristic curve
IDI: integrated discrimination improvement index
NRI: net reclassification index
OR: Odds ratio
CI: Confidence interval
BMI: Body mass index
ICU: Intensive care unit
ECMO: extracorporeal membrane oxygenation
CRP: C-reactive protein
PaO_2_/FiO_2_: arterial oxygen tension/inspired oxygen fraction
DCD: Donation after Circulatory Death
DBD: Donation after Brain Death
IQR: Interquartile range
LAR: lactate-to-albumin ratio
LTx: lung transplantation
RSS: Risk Stratification Score
PNI: Prognostic Nutritional Index
PGD3: primary graft dysfunction grade 3
DCA: Decision curve analysis
NB: Net benefit
AIC: smallest Akaike information criterion
VIF: variance inflation factor
EPV: events per variable
MICE: multiple imputation by chained equations
