# Supplementary material for: Lactate-to-albumin ratio improves prediction of perioperative mortality in lung transplant patients
Source: Front Med (Lausanne). 2026 Aug 18;13:1837904. doi: 10.3389/fmed.2026.1837904 (PMC13548395; doi:10.3389/fmed.2026.1837904)
Supplement: Supplementary file 1 [file Supplementary_file_1.doc]

**Supplemental materials**

Figure S1. Flow chart of the study.

Figure S2. Distribution of missing variables.

Figure S3. The Nonlinear Association Between Surgery Time and the Log Odds of 30-Day Mortality.

Figure S4. Correlation analysis between variables.

Figure S5. Analysis of the linear relationship between continuous independent variable and LogitP.

Table S1Variance inflation factors (VIFs) for variables in the modified model.

Table S2. Model performance metrics for base model and modified model across multiple imputed datasets.

Supplementary Statistical Analysis

Figure S1. Flow chart of the study.

Figure S2. Distribution of missing variables.

CRP, C-reactive protein; PaO2/FiO2, arterial oxygen tension/inspired oxygen fraction; ECMO, extracorporeal membrane oxygenation; BMI, Body mass index;

Figure S3. The Nonlinear Association Between Surgery Time and the Log Odds of 30-Day Mortality.

Figure S4. Correlation analysis between variables. Color intensity represents the strength of correlation (blue = negative correlation, red = positive correlation), with values indicating the Pearson correlation coefficient (r).

|  |  |
| --- | --- |
|  |  |
| Figure S5. Analysis of the linear relationship between continuous independent variable and LogitP. | |

| Table S1. Variance inflation factors (VIFs) for variables in the modified model. | |
| --- | --- |
| Variables | VIF |
| Fluid balance(l) | 1.57 |
| Cold ischemia time (h) | 1.04 |
| Blood transfusion (u) | 2.17 |
| Lactate-to-albumin ratio | 1.60 |
| VIF values below 5 indicate minimal risk of multicollinearity. | |

| Table S2. Model performance metrics for base model and modified model across multiple imputed datasets. | | | | | | | | |
| --- | --- | --- | --- | --- | --- | --- | --- | --- |
| **Dataset** | **Model** | **AUC (95% CI)** | **DeLong P-value** | **Corrected AUC** | **Brier Score** | **Corrected Brier Score** | **Hosmer-Lemeshow P-value** |  |
| Imputation dataset (1) | Base model | 0.764 (0.691–0.836) | - | 0.749 | 0.103 | 0.106 | 0.697 |  |
|  | Modified model | 0.807 (0.741–0.873) | 0.024 | 0.794 | 0.093 | 0.097 | 0.544 |  |
| Imputation dataset (2) | Base model | 0.764 (0.691–0.836) | - | 0.751 | 0.103 | 0.106 | 0.697 |  |
|  | Modified model | 0.807 (0.741–0.873) | 0.024 | 0.795 | 0.093 | 0.097 | 0.544 |  |
| Imputation dataset (3) | Base model | 0.764 (0.691–0.836) | - | 0.747 | 0.103 | 0.107 | 0.592 |  |
|  | Modified model | 0.807 (0.741–0.873) | 0.024 | 0.793 | 0.093 | 0.098 | 0.544 |  |
| Imputation dataset (4) | Base model | 0.763 (0.690–0.836) | - | 0.753 | 0.103 | 0.106 | 0.697 |  |
|  | Modified model | 0.807 (0.741–0.873) | 0.024 | 0.795 | 0.093 | 0.097 | 0.544 |  |
| Imputation dataset (5) | Base model | 0.764 (0.691–0.837) | - | 0.75 | 0.103 | 0.106 | 0.698 |  |
|  | Modified model | 0.807 (0.741–0.873) | 0.024 | 0.793 | 0.093 | 0.098 | 0.544 | |
| AUC: Area under the receiver operating characteristic curve; CI: Confidence interval. | | | | | | | | |
| P-values based on DeLong test. | | | | | | | | |
| Corrected values refer to bootstrap-adjusted estimates. | | | | | | | | |

**Supplementary Statistical Analysis**

Demographic, preoperative, intraoperative, postoperative, and donor characteristics were analyzed. The median (interquartile range) is used to describe continuous variables, while categorical variables are represented by counts and percentages. Missing data were addressed using the multiple imputation by chained equations (MICE) method with the mice package in R.[14] Five imputed datasets were generated, and models were developed for each dataset. The linear relationship between continuous variables and the outcome was evaluated graphically using restricted cubic splines (RCS), with non-linear variables subsequently categorized. Continuous variables were analyzed using t-tests or Mann–Whitney U tests, depending on their distribution. Categorical variables were analyzed using the χ2 test or Fisher’s exact test, based on the expected number of subjects.

Univariate logistic regression was conducted to identify potential predictors of perioperative mortality. Variables with statistical significance (p < 0.05) in univariate analyses were included in a multivariate logistic regression model. The base model was constructed using bidirectional stepwise regression, with selection criteria based on the smallest Akaike information criterion (AIC). Two models were developed: a base model (excluding LAR) and a modified model (incorporating LAR) for predicting perioperative mortality. To validate the assumptions of the multivariate logistic regression model, RCS analysis was used to assess the linearity between specific continuous independent variables and the logit of the outcome, adjusting for other model variables. Multicollinearity was assessed using the variance inflation factor (VIF), such that VIF values lower than 5 typically indicate low multicollinearity. Adjusted odds ratios (ORs) and 95% confidence intervals (CIs) for the variables in the modified model were reported. A rule based on a ratio of 10 events per variable (EPV) was used to avoid over-fitting. The discriminatory power of the predictive models was assessed using the area under the receiver operating characteristic curve (AUC). An AUC of 0.5 indicates no discrimination, whereas an AUC ≥ 0.7 suggests moderate discrimination. Model performance improvements between the base and modified models were evaluated by comparing AUCs using the DeLong test. Model fit was assessed using the likelihood ratio test. Calibration was evaluated through Brier scores, calibration curves, and the Hosmer–Lemeshow (H–L) goodness-of-fit test. Calibration curves were plotted to compare predicted probabilities against observed outcome frequencies. The closer the calibration curve aligns with the diagonal line, the better the model's calibration. A Brier score exceeding 0.25 suggests inadequate calibration, while a non-significant H–L test p-value (p > 0.05) indicated satisfactory model fit. Internal validation of the predictive models was performed using the bootstrapping method to mitigate overestimation of AUC in performance evaluation.[15] Bootstrap resampling involved drawing random samples, with replacement, from the original dataset to create multiple simulated samples. Bias-corrected performance measures were obtained by calculating the difference between bootstrap performance and test performance across 1000 repetitions, and adjusting the apparent performance accordingly. A bias-corrected AUC was calculated using 1000 bootstrap resamples to evaluate discrimination ability, and a bias-corrected calibration curve was generated to assess calibration.

Decision curve analysis (DCA) was performed to evaluate the clinical value of prediction model by measuring their net benefit at various probability thresholds. The net benefit (NB) represents the balance between true positives and false positives. A model demonstrates clinical usefulness if its net benefit curve exceeds those of the "full treatment" or "no treatment" strategies. Quantitative improvements in predictive performance resulting from the incorporation of LAR into the base model were evaluated using Continuous Net Reclassification Improvement (NRI) and Integrated Discrimination Improvement (IDI). Positive values for NRI and IDI indicate enhanced discriminative ability of the modified model compared to the base model. The modified model was subsequently transformed into a nomogram based on the results of the multivariable logistic regression analysis. The nomogram predicts perioperative mortality risk by calculating a total score derived from individual predictor contributions, with the total score corresponding to the estimated probability of the outcome.

All statistical analyses were conducted using RStudio (version 4.3.3). The analysis utilized the R packages "rms," "mice," "table1," "ggplot2," "riskRegression," "PredictABEL," "ResourceSelection," "plotROC," and "dcurves." Two-sided tests were employed, and a p-value of < 0.05 was considered statistically significant.
